# The combination of chest compression synchronized ventilation and aortic balloon occlusion improve the outcomes of cardiopulmonary resuscitation in swine

**DOI:** 10.3389/fmed.2022.1057000

**Published:** 2022-12-21

**Authors:** Jiefeng Xu, Zafar Ullah Khan, Minhai Zhang, Jiangang Wang, Meiya Zhou, Zhongjun Zheng, Qijiang Chen, Guangju Zhou, Mao Zhang

**Affiliations:** ^1^Department of Emergency Medicine, The Second Affiliated Hospital, Zhejiang University School of Medicine, Hangzhou, China; ^2^Key Laboratory of the Diagnosis and Treatment of Severe Trauma and Burn of Zhejiang Province, Hangzhou, China; ^3^Zhejiang Provincial Clinical Research Center for Emergency and Critical Care Medicine, Hangzhou, China; ^4^Hangzhou Emergency Medical Center, Hangzhou, China; ^5^Department of Intensive Care Medicine, The First Hospital of Ninghai, Ningbo, China

**Keywords:** aortic balloon occlusion, cardiac arrest, cardiopulmonary resuscitation, chest compression synchronized ventilation, hemodynamics, oxygenation, organ protection

## Abstract

**Aim:**

The primary mission of cardiopulmonary resuscitation (CPR) is to provide adequate blood flow and oxygen delivery for restoring spontaneous circulation from cardiac arrest (CA) events. Previously, studies demonstrated that chest compression synchronized ventilation (CCSV) improved systemic oxygen supply during CPR, and aortic balloon occlusion (ABO) augments the efficacy of external CPR by increasing blood perfusion to vital organs. However, both them failed to make a significant improvement in return of spontaneous circulation (ROSC). In this study, we investigated the effects of combined CCSV and ABO on the outcomes of CPR in swine.

**Methods:**

Thirty-one male domestic swine were subjected to 8 min of electrically induced and untreated CA followed by 8 min of CPR. CPR was performed by continuous chest compressions and mechanical ventilation. At the beginning of CPR, the animals were randomized to receive intermittent positive pressure ventilation (IPPV, *n* = 10), CCSV (*n* = 7), IPPV + ABO (*n* = 7), or CCSV + ABO (*n* = 7). During CPR, gas exchange and systemic hemodynamics were measured, and ROSC was recorded. After resuscitation, the function and injury biomarkers of vital organs including heart, brain, kidney, and intestine were evaluated.

**Results:**

During CPR, PaO_2_ was significantly higher accompanied by significantly greater regional cerebral oxygen saturation in the CCSV and CCSV + ABO groups than the IPPV group. Coronary perfusion pressure, end-tidal carbon dioxide, and carotid blood flow were significantly increased in the IPPV + ABO and CCSV + ABO groups compared with the IPPV group. ROSC was achieved in five of ten (IPPV), five of seven (CCSV), six of seven (IPPV + ABO), and seven of seven (CCSV + ABO) swine, with the rate of resuscitation success being significantly higher in the CCSV + ABO group than the IPPV group (*P* = 0.044). After resuscitation, significantly improved myocardial and neurological function, and markedly less cardiac, cerebral, renal, and intestinal injuries were observed in the CCSV + ABO group compared with the IPPV group.

**Conclusion:**

The combination of CCSV and ABO improved both ventilatory and hemodynamic efficacy during CPR, promoted ROSC, and alleviated post-resuscitation multiple organ injury in swine.

## Introduction

Incidence and mortality of cardiac arrest (CA) remain alarmingly high worldwide. It is estimated that 44.2–135.5 individuals per 100, 000 population in United States, and 67–170 individuals per 100, 000 population in Europe experience out-of-hospital CA events annually; however, their survival to hospital discharge are on average 9.0 and 8.0%, respectively (1, 2). In China, the estimated amount of out-of-hospital CA victims was up to 550, 000 and its survival rate was less than 1% every year (3). After experiencing CA events, rapid initiation of cardiopulmonary resuscitation (CPR) and its high-quality performance are considered to be most important for saving the life of CA victims (4). Although the advances in CPR theory, technique, and training have gradually improved (5); most victims still fail to achieve return of spontaneous circulation (ROSC) from CA events, or suffer from severe post-resuscitation myocardial and neurological dysfunction, which result in a significant in-hospital mortality (1, 6). Currently, the strategy of CPR performance remains to be further improved.

The optimization of oxygenation and perfusion during CPR performance are the key factors for restoring spontaneous circulation in CA victims. Previously, two investigations demonstrated that chest compression synchronized ventilation (CCSV), a novel mode of pressure-controlled ventilation triggered by each chest compression, could improve gas exchange and therefore increase blood oxygenation during CPR in swine (7, 8). Recently, three investigations demonstrated that aortic balloon occlusion (ABO), used as one technique to rapidly increase aortic blood blow to proximal organ while simultaneously decrease its perfusion to distal organ, could improve cardiac and cerebral blood circulation during CPR in swine (9–11). However, both of these two techniques did not make a significant improvement in ROSC in these studies.

Currently, it is worthy to confirm that whether the combination of CCSV and ABO implemented during CPR can simultaneously augment oxygen supply and blood perfusion so as to further improve the efficacy of CPR. The present study was designed to investigate the effects of combined CCSV and ABO on the outcomes of CPR after CA events in a clinically relevant swine model. We hypothesized that the combination of CCSV and ABO would improve ventilatory and hemodynamic parameters during CPR, and further resulted in the increase of ROSC and the alleviation of post-resuscitation multiple organ injury in a swine model of CA and resuscitation.

## Materials and methods

Thirty-one healthy male white domestic swine, aged 4–6 months, weighing 38 ± 3 kg, were supplied by Shanghai Jiagan Biotechnology Inc. (Shanghai, China). The animals were fed under the standard conditions including standard atmospheric pressure, room temperature (20–25°C), humidity (60–80%), 12/12 h light/dark cycle, closed cage, spontaneous water intake, regular eating, regular cleaning, and disinfection. All animals received humane care in compliance with the Institutional Animal Care and Use Committee guidelines. The study was approved by the Institutional Animal Care and Use Committee of the Second Affiliated Hospital, Zhejiang University School of Medicine (approval number: 2020–15).

### Animal preparation

The animals were sedated by intramuscular midazolam (0.4–0.5 mg/kg), and then anesthetized by intravenous propofol (2 mg/kg) followed by a continuous infusion of propofol (4 mg/kg/h). They were intubated and ventilated with an emergency and transport ventilator (MEDUMAT Standard 2, Weinmann GmbH + Co.KG, Hamburg, Germany) with an initial setting of tidal volume (10 ml/kg), respiratory rate (12/min), I:E ratio (1:2), upper airway pressure limit (60 mbar), and FiO_2_ (21%). End-tidal CO_2_ (ETCO_2_) and electrocardiogram were monitored by a monitor/defibrillator (M Series, ZOLL Medical Corporation, Chelmsford, MA), and a patient monitoring system (BeneVision N22, Mindray, Shenzhen, China).

Two 7-French thermodilution catheters were inserted from the right femoral artery and vein into the thoracic aorta and right atrium, in which their positions were confirmed by characteristic pressure morphology and with fluoroscopy, and then connected to the patient monitoring system for the measurements of aortic and right atrial pressures. One 4-French thermistor-tipped arterial catheter *via* an 8-French artery sheath and another 7-French central venous catheter were inserted into the left femoral artery and right internal jugular vein, and then connected to the PiCCO system (PiCCOplus, Pulsion Medical Systems, Munich, Germany) for the measurements of stroke volume (SV) and global ejection fraction (GEF). One 5-French pacing catheter was inserted from the right external jugular vein into the right ventricle, and then used to induce CA. One flow probe of ultrasonic blood flow meter (T402, Transonic Systems, Ithaca, NY) was placed around right common carotid artery to measure carotid blood flow (CBF). The probe of cerebral oxygen monitor (EGOS-600B, Suzhou Engin Bio-medical Electronics Co., Suzhou, China) was placed over the forehead to measure regional cerebral oxygen saturation (rSO_2_).

### Experimental procedures

After the animals were stabilized for 15 min and baseline measurements were obtained, CA was induced by 1 mA alternating current delivered to right ventricular endocardium. The ventilation was stopped and the pacing catheter was removed. After 8 min of untreated CA, CPR was performed with manual chest compression and mechanical ventilation. Chest compressions were continuously provided by two professional providers, and the compression quality was monitored using a feedback device (PalmCPR, Sunlife, Shanghai, China).

At the beginning of CPR, the animals were randomized by the sealed envelope method to receive intermittent positive pressure ventilation (IPPV, *n* = 10), CCSV (*n* = 7), IPPV + ABO (*n* = 7), or CCSV + ABO (*n* = 7). In the IPPV and IPPV + ABO groups, the ventilation was implemented with the setting of tidal volume (7 ml/kg), respiratory rate (10/min), I:E ratio (1:2), upper airway pressure limit (60 mbar), and FiO_2_ (100%). In the CCSV and CCSV + ABO groups, the ventilation was triggered by each chest compression with the setting of inspiratory pressure (60 mbar), inspiratory time (205 ms), and FiO_2_ (100%). In the IPPV + ABO and CCSV + ABO groups, one 7-French aortic balloon catheter (Cordis Corporation, Hialeah, FL) was inflated during CPR to stop distal flow of arterial blood. This catheter was placed during CA, in which the 4-French thermistor-tipped arterial catheter was withdrawn from the left femoral artery sheath, and then aortic balloon catheter was inserted from this artery sheath into descending thoracic aorta. The position of aortic balloon was located at the level of the diaphragm using surface landmark and with fluoroscopy. A complete occlusion was performed by insufflating the balloon to reach six atmospheric pressures according to the manufacturer’s instruction.

After 2 min of CPR, a dose of 20 μg/kg of epinephrine was given, and then the same dose was administered every 4 min. After 8 min of CPR, a single 150-J biphasic electrical shock was delivered by the monitor/defibrillator. If an organized rhythm was observed and a mean arterial pressure (MAP) >50 mm Hg was maintained for 5 min or more, the animals was considered to obtain ROSC. If not, CPR was immediately restarted for 2 min prior to another electrical shock. This protocol was repeated until ROSC or for a total of 18 min (12, 13). Following ROSC, the animals were monitored for 4 h, then intravascular catheters were removed, and thereafter the propofol was stopped. Once the animals recovered from the anesthesia, they were extubated and then observed in their cages for an additional 20 h. After that, the animals were euthanized with an intravenous injection of propofol (3 mg/kg), followed by 10 ml of potassium chloride (10%). A necropsy was performed to record those possible injuries resulting from the surgical or CPR intervention or the presence of obfuscating diseases.

### Measurements

Heart rate (HR), MAP, ETCO_2_, CBF, and rSO_2_ were continuously monitored throughout the experiment. During CPR, coronary perfusion pressure (CPP) was calculated as the differences between decompression diastolic aortic and right atrial pressures. Arterial blood gas and lactate concentrations were measured at baseline, 4 and 7 min of CPR, and 1, 2, and 4 h after resuscitation using a Blood Gas Analyzer (iSTAT300, Abbott, Chicago, IL).

Myocardial function, as evaluated by the changes of SV and GEF, was measured at baseline, and 1, 2, and 4 h after resuscitation. Cardiac, cerebral, renal, and intestinal injury biomarkers including cardiac troponin I (cTnI), neuron specific enolase (NSE), S100B protein (S100B), creatinine (Cr), and intestinal fatty acid binding protein (IFABP) were measured at baseline, and 1, 2, 4, and 24 h after resuscitation. For this purpose, venous blood samples were collected to measure their serum levels with enzyme-linked immunosorbent assay kits (Meixuan Biotechnology Inc., Shanghai, China) according to the manufacturer’s instruction. Neurologic function was evaluated using the method of neurological deficit score (NDS) at 24 h after resuscitation, in which the NDS was scored from 0 (no neurologic deficit) to 400 (death or brain death) (14), and examined by two investigators who were blinded to the allocations.

Cardiac, cerebral, renal, and intestinal tissue samples were obtained at 24 h after resuscitation, and their pathological analysis was performed by an experienced pathologist who was blinded to the allocations. Left ventricular myocardium, cerebral cortex, hippocampus, left kidney, and distal ileum were harvested immediately after animal euthanasia. Tissue specimens were fixed in 4% paraformaldehyde for 24 h, embedded in paraffin, and cut in a 5-mm section. The sections were stained with TdT-mediated dUTP nick end labeling (TUNEL) assay kits (Boster Biological Technology Co., Ltd., Wuhan, China) according to the manufacturer’s instruction. Six views were randomly chosen in each section to count the numbers of TUNEL-positive cells and total cells at 200 × magnification under an optical microscope (biological microscope CX31, Olympus, Japan). The rate of apoptotic cells was calculated as the percentage of TUNEL-positive cells/total cells.

### Statistical analysis

The distribution of continuous variables was confirmed with the Kolmogorov–Smirnov test. If the data were normally distributed, they were presented as mean ± standard deviation and compared with one way analysis of variance. If the data were not normally distributed, they were presented as a median (25–75th percentiles) and compared with the Kruskal–Wallis test. Comparisons between time-based measurements within each group were performed with repeated-measurement analysis of variance. If there was a significant difference in the overall comparison of groups, comparisons between any other two groups were made by the Bonferroni test. For the comparisons of categorical variables including the rates of ROSC, 4-h and 24-h survival, the data were compared with the Fisher’s exact test. A value of *P* < 0.05 was considered significant.

## Results

Thirty-one studies were performed and finished. There were no significant differences in baseline hemodynamics, myocardial function, arterial blood gas and lactate, and venous organ injury biomarkers among the four groups (Tables 1, 2).

**TABLE 1:** Baseline characteristics.

Groups	IPPV	CCSV	IPPV + ABO	CCSV + ABO
Body weight (kg)	38 ± 3	38 ± 2	39 ± 3	39 ± 2
Heart rate (beats/min)	98 ± 9	95 ± 5	91 ± 5	91 ± 5
Mean arterial pressure (mmHg)	112 ± 8	112 ± 7	114 ± 7	112 ± 6
End-tidal carbon dioxide (mmHg)	41 ± 2	40 ± 3	40 ± 2	41 ± 1
Carotid blood flow (ml/min)	206 ± 17	210 ± 20	206 ± 15	206 ± 23
Regional cerebral oxygen saturation (%)	59 ± 2	59 ± 1	58 ± 3	61 ± 3
Stroke volume (ml)	79 ± 8	79 ± 8	75 ± 4	80 ± 6
Global ejection fraction (%)	35 ± 5	35 ± 5	32 ± 6	37 ± 5
Cardiac troponin I (pg/ml)	168 ± 33	168 ± 10	179 ± 25	177 ± 31
Neuron specific enolase (ng/ml)	19.7 ± 1.8	18.7 ± 1.4	17.6 ± 1.9	17.9 ± 2.1
S100B protein (pg/ml)	1,509 ± 155	1,542 ± 233	1,474 ± 167	1,495 ± 280
Creatinine (μmol/L)	74 ± 10	69 ± 22	74 ± 11	69 ± 18
Intestinal fatty acid binding protein (μg/L)	469 ± 30	456 ± 26	451 ± 35	474 ± 55

IPPV, intermittent positive pressure ventilation; CCSV, chest compression synchronized ventilation; ABO, aortic balloon occlusion.

Values are presented as mean ± standard deviation. Each group contained 7–10 swine at baseline.

**TABLE 2:** Arterial blood gas and lactate.

	Baseline	Cardiopulmonary resuscitation		Post-resuscitation		
		4 minute	7 minute	1 hour	2 hour	4 hour
**pH**						
IPPV	7.43 ± 0.06	7.33 ± 0.10	7.31 ± 0.12	7.25 ± 0.19	7.39 ± 0.07	7.47 ± 0.03
CCSV	7.42 ± 0.06	7.55 ± 0.10^a^	7.48 ± 0.12^a^	7.32 ± 0.03	7.38 ± 0.02	7.45 ± 0.01
IPPV + ABO	7.45 ± 0.07	7.33 ± 0.14^b^	7.27 ± 0.16^b^	7.34 ± 0.03	7.37 ± 0.10	7.42 ± 0.12
CCSV + ABO	7.43 ± 0.02	7.49 ± 0.14^a^	7.46 ± 0.18	7.34 ± 0.06	7.43 ± 0.05	7.50 ± 0.01^b^
**PaCO 2 (mm Hg)**						
IPPV	45 ± 5	38 ± 7	37 ± 9	47 ± 8	40 ± 1	38 ± 5
CCSV	45 ± 6	23 ± 4^a^	25 ± 11^a^	40 ± 3	42 ± 3	42 ± 2
IPPV + ABO	41 ± 7	42 ± 13^b^	37 ± 11	41 ± 5	44 ± 9	41 ± 3
CCSV + ABO	45 ± 5	30 ± 12	28 ± 12	44 ± 9	40 ± 4	39 ± 3
**PaO 2 (mm Hg)**						
IPPV	76 ± 8	115 ± 44	107 ± 51	55 ± 23	66 ± 14	59 ± 8
CCSV	80 ± 5	433 ± 61^a^	466 ± 69^a^	75 ± 17	69 ± 13	67 ± 14
IPPV + ABO	74 ± 12	105 ± 58^b^	103 ± 78^b^	65 ± 8	65 ± 12	65 ± 18
CCSV + ABO	74 ± 10	372 ± 123^ac^	412 ± 107^ac^	64 ± 4	67 ± 7	73 ± 6^a^
**Base excess**						
IPPV	5.6 ± 3.5	−6.0 ± 2.1	−8.3 ± 2.3	−4.2 ± 6.3	−0.2 ± 5.1	4.0 ± 2.1
CCSV	4.7 ± 3.6	−2.5 ± 3.9^a^	−6.1 ± 4.5	−4.5 ± 3.4	−0.3 ± 1.7	4.3 ± 0.5
IPPV + ABO	4.7 ± 5.5	−3.6 ± 3.0	−7.0 ± 3.3	−4.0 ± 2.3	0.0 ± 3.7	3.5 ± 6.0
CCSV + ABO	4.9 ± 2.7	−2.3 ± 3.8^a^	−5.0 ± 5.0	−2.3 ± 3.5	2.9 ± 3.1	6.1 ± 2.0
**Lactate (mmol/L)**						
IPPV	1.2 ± 0.7	8.4 ± 1.3	9.5 ± 0.9	8.6 ± 3.8	6.3 ± 3.2	2.7 ± 0.8
CCSV	1.3 ± 0.4	6.9 ± 1.9	9.2 ± 1.1	8.2 ± 1.2	4.7 ± 0.5	1.9 ± 0.3
IPPV + ABO	1.5 ± 0.6	6.9 ± 1.3^a^	9.3 ± 1.3	7.0 ± 2.6	4.4 ± 2.7	1.8 ± 1.0
CCSV + ABO	1.5 ± 0.6	6.7 ± 1.5^a^	8.7 ± 1.2	6.1 ± 1.6	3.1 ± 1.5	1.4 ± 0.7^a^

IPPV, intermittent positive pressure ventilation; CCSV, chest compression synchronized ventilation; ABO, aortic balloon occlusion.

Values are presented as mean ± standard deviation. ^a^*P* < 0.05 vs. IPPV group; ^b^*P* < 0.05 vs. CCSV group; ^c^*P* < 0.05 vs. IPPV + ABO group.

Each group contained 7–10 swine at baseline and during cardiopulmonary resuscitation, and 4–7 swine after resuscitation, respectively.

**FIGURE 1:**
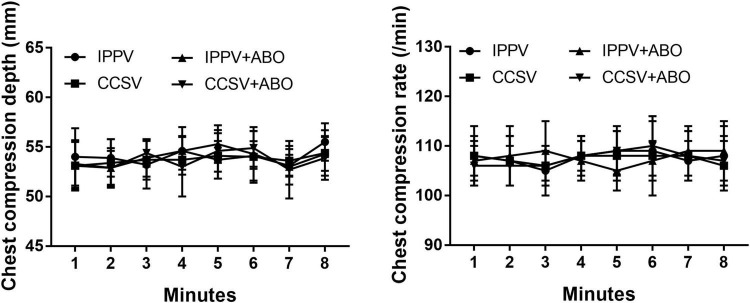
The changes of chest compression depth and rate during cardiopulmonary resuscitation (CPR). IPPV, intermittent positive pressure ventilation; CCSV, chest compression synchronized ventilation; ABO, aortic balloon occlusion. Each group contained 7–10 swine during CPR.

**FIGURE 2:**
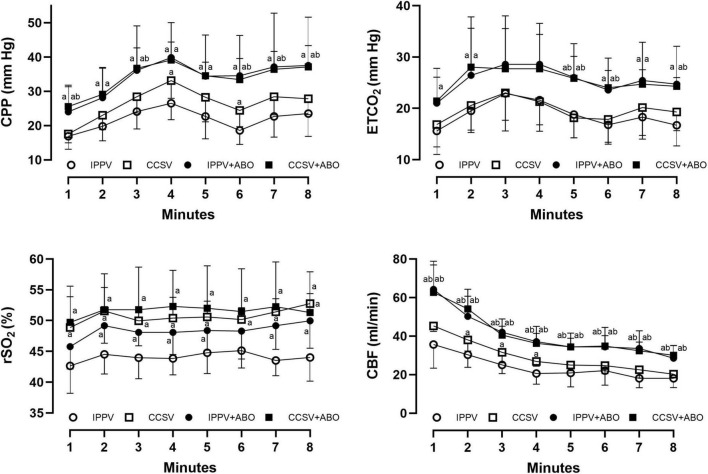
The changes of coronary perfusion pressure (CPP), end-tidal carbon dioxide (ETCO_2_), regional cerebral oxygen saturation (rSO_2_), and carotid blood flow (CBF) during cardiopulmonary resuscitation (CPR). IPPV, intermittent positive pressure ventilation; CCSV, chest compression synchronized ventilation; ABO, aortic balloon occlusion. Each group contained 7–10 swine during CPR. ^a^*P* < 0.05 for CCSV + ABO group, IPPV + ABO group, CCSV group vs. IPPV group; ^b^*P* < 0.05 for CCSV + ABO group (right b), IPPV + ABO group (left b) vs. CCSV group.

During CPR, the same levels of chest compression depth and rate were achieved by our professional providers, in which no difference in CPR quality was observed among the four groups (Figure 1). However, significantly higher levels of CPP, ETCO_2_, and CBF during CPR were obtained in the IPPV + ABO and CCSV + ABO groups than those in the IPPV and CCSV groups (all *P* < 0.05, Figure 2). In addition, the similar level of rSO_2_ was observed during CPR in the CCSV and CCSV + ABO groups, which was significantly greater than that in the IPPV group (all *P* < 0.05, Figure 2). The level of rSO_2_ tended to be higher in these two groups than the IPPV + ABO group although the difference was not statistically significant (Figure 2).

During CPR, the increases in PaO_2_ and pH and the decrease in PaCO_2_ were observed in the CCSV and CCSV + ABO groups: The values of PaO_2_ were significantly greater in these two groups than the IPPV and IPPV + ABO groups, and the values of pH were significantly higher and the values of PaCO_2_ were markedly lower in the CCSV group than the IPPV and IPPV + ABO groups (all *P* < 0.05, Table 2). Base excess was decreased and lactate was increased during CPR in all groups; however, the changes in them were milder in the CCSV, IPPV + ABO, and CCSV + ABO groups than the IPPV group (Table 2).

A higher rate of ROSC was achieved in the CCSV + ABO group than the other three groups, and the difference was statistically significant between the CCSV + ABO and IPPV groups (*P* < 0.05, Table 3). Duration of CPR, epinephrine dosage, and numbers of defibrillation that were required for establishing ROSC were significantly smaller in the CCSV + ABO group than the IPPV group (all *P* < 0.05, Table 3). The rates of 4-h and 24-h survival after resuscitation were also significantly higher in the CCSV + ABO group compared with the IPPV group (both *P* < 0.05, Table 3).

**TABLE 3:** Cardiopulmonary resuscitation outcomes and survival.

Groups	IPPV	CCSV	IPPV + ABO	CCSV + ABO
Duration of CPR (min)	13.2 ± 5.1	11.4 ± 4.6	9.7 ± 3.7	8.0 ± 0.0^ab^
Epinephrine dosage (mg)	2.8 ± 1.3	2.4 ± 1.0	2.0 ± 0.9	1.6 ± 0.1^a^
Numbers of defibrillation (*n*)	3.6 ± 2.5	2.7 ± 2.3	1.9 ± 1.9	1.0 ± 0.0^a^
Return of spontaneous circulation (n/n)	5/10	5/7	6/7	7/7^a^
4-h survival rate (n/n)	5/10	4/7	6/7	7/7^a^
24-h survival rate (n/n)	5/10	4/7	5/7	7/7^a^

IPPV, intermittent positive pressure ventilation; CCSV, chest compression synchronized ventilation; ABO, aortic balloon occlusion.

Values are presented as mean ± standard deviation. ^a^*P* < 0.05 vs. IPPV group; ^b^*P* < 0.05 vs. CCSV group. Each group contained 7–10 swine during cardiopulmonary resuscitation and 4–7 swine after resuscitation, respectively.

**FIGURE 3:**
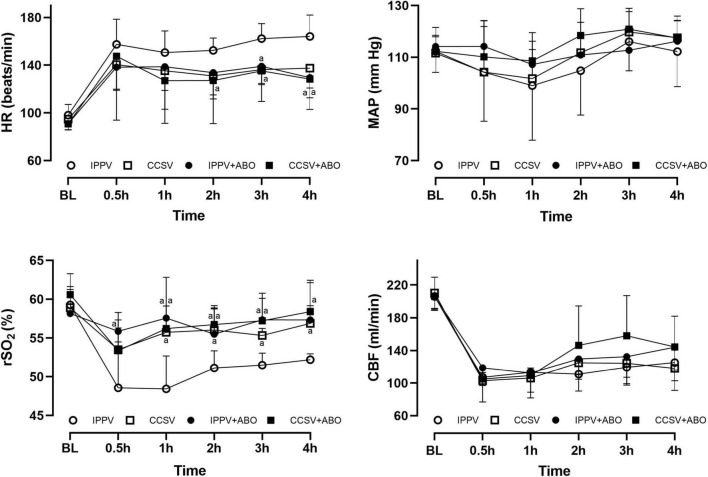
The changes of heart rate (HR), mean arterial pressure (MAP), regional cerebral oxygen saturation (rSO_2_), and carotid blood flow (CBF) at baseline (BL) and after resuscitation. IPPV, intermittent positive pressure ventilation; CCSV, chest compression synchronized ventilation; ABO, aortic balloon occlusion. Each group contained 7–10 swine at BL, and 4–7 swine after resuscitation, respectively. ^a^*P* < 0.05 for CCSV + ABO group, IPPV + ABO group, CCSV group vs. IPPV group.

After resuscitation, the increase in HR and the decrease in MAP, CBF, and rSO_2_ were observed in all survived animals. However, HR decreased more quickly in the CCSV + ABO group than the other three groups, with the differences 2 h after resuscitation being statistically significant between the CCSV + ABO and IPPV groups (all *P* < 0.05, Figure 3). Post-resuscitation MAP and CBF increased more quickly in the CCSV + ABO group than the other three groups although the difference was not statistically significant. The values of rSO_2_ were maintained at a higher level in the CCSV, IPPV + ABO, and CCSV + ABO groups, which were significantly greater than that in the IPPV group (all *P* < 0.05, Figure 3).

After resuscitation, the lactate decreased more rapidly in the CCSV + ABO group than the other three groups, with the difference at 4 h post-resuscitation being significant between the CCSV + ABO and IPPV groups (*P* < 0.05, Table 2). No significant differences were observed in post-resuscitation pH, PaCO_2_, PaO_2_, and base excess among the four groups (Table 2).

**FIGURE 4:**
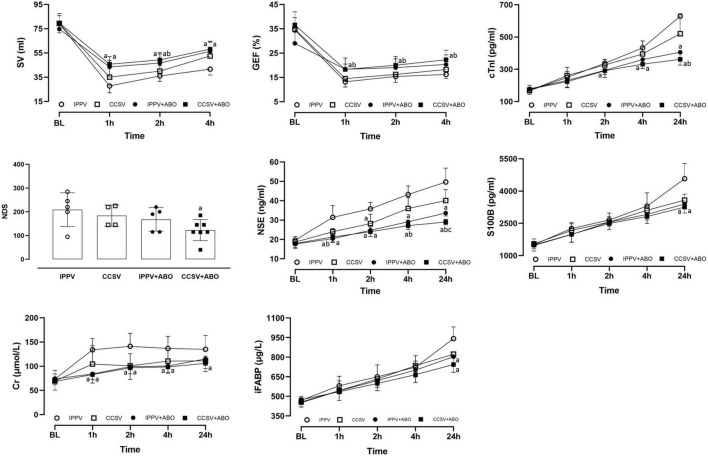
The changes of stroke volume (SV), global ejection fraction (GEF), cardiac troponin I (cTnI), neurological deficit score (NDS), neuron specific enolase (NSE), S100B protein (S100B), creatinine (Cr), and intestinal fatty acid binding protein (IFABP) at baseline (BL) and after resuscitation. IPPV, intermittent positive pressure ventilation; CCSV, chest compression synchronized ventilation; ABO, aortic balloon occlusion. Each group contained 7–10 swine at BL, and 4–7 swine after resuscitation, respectively. ^a^*P* < 0.05 for CCSV + ABO group, IPPV + ABO group, CCSV group vs. IPPV group; ^b^*P* < 0.05 for CCSV + ABO group (right and inferolateral b), IPPV + ABO group (left b) vs. CCSV group; ^c^*P* < 0.05 for CCSV + ABO group vs. IPPV + ABO group.

After resuscitation, myocardial function indicated by the changes of SV and GEF was significantly impaired in all survived animals when compared with the baseline value; however, the values of SV were significantly greater in the IPPV + ABO and CCSV + ABO groups than the IPPV group, and the values of GEF were significantly higher in the CCSV + ABO group than the IPPV and CCSV groups (all *P* < 0.05, Figure 4). After resuscitation, neurological dysfunction evaluated by the score of NDS was observed in all groups; however, NDS was better in the CCSV + ABO group than the other three groups, with the difference being significant between the CCSV + ABO and IPPV groups (*P* < 0.05, Figure 4).

After resuscitation, the serum levels of cTnI, NSE, S100B, Cr, and IFABP indicating cardiac, cerebral, renal, and intestinal injuries were increased in all survived animals. However, the serum levels of NSE (1, 2, 4, and 24 h), Cr (1, 2, and 4 h), cTnI (4 and 24 h), S100B (24 h), and IFBAP (24 h) after resuscitation were significantly lower in the IPPV + ABO and CCSV + ABO groups than the IPPV group (all *P* < 0.05, Figure 4). The serum levels of NSE (4 and 24 h) and cTnI (24 h) after resuscitation were significantly less in the CCSV + ABO group than the CCSV group (all *P* < 0.05, Figure 4). The serum levels of NSE (24 h) after resuscitation was significantly decreased in the CCSV + ABO group compared with the IPPV + ABO group (*P* < 0.05, Figure 4).

**FIGURE 5:**
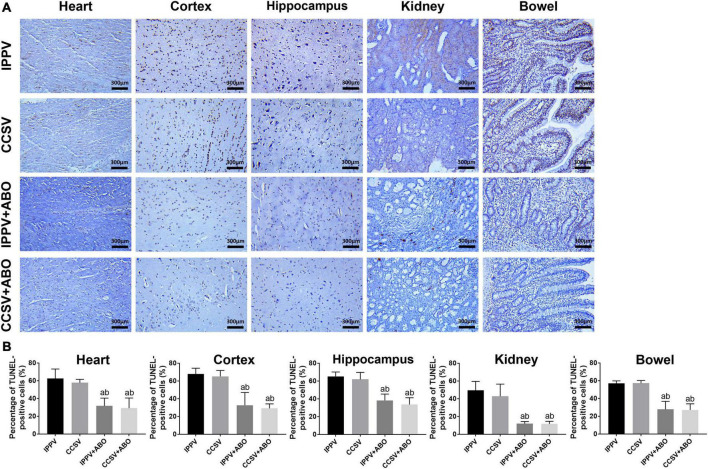
The evaluation of pathological damage in the heart, cortex, hippocampus, kidney, and intestine at 24 h post-resuscitation among the four groups. **(A)** Representative photomicrographs of terminal deoxynucleotidyl transferase dUTP nick-end labeling (TUNEL) assay (200 × magnification). **(B)** The percentage of TUNEL-positive cells. IPPV, intermittent positive pressure ventilation; CCSV, chest compression synchronized ventilation; ABO, aortic balloon occlusion. Tissue samples were obtained at 24 h post-resuscitation, in which each group contained 4–6 swine. ^a^*P* < 0.05 for CCSV + ABO group, IPPV + ABO group vs. IPPV group; ^b^*P* < 0.05 for CCSV + ABO group, IPPV + ABO group vs. CCSV group.

At 24 h post-resuscitation, the necropsy did not show any procedure related complications in all groups. However, the pathologic damage occurred in the heart, cortex, hippocampus, kidney, and intestine in all of them. The percentage of TUNEL-positive cells were significantly decreased in the IPPV + ABO and CCSV + ABO groups compared with the IPPV and CCSV groups (all *P* < 0.05, Figure 5), and also less in the CCSV + ABO group than the IPPV + ABO group although the difference was not statistically significant (Figure 5).

## Discussion

The present study demonstrated that during CPR, PaO_2_ and rSO_2_ were significantly increased in those animals ventilated with the CCSV mode, and CPP, ETCO_2_, and CBF were significantly elevated in those animals receiving the ABO when compared with the IPPV group. A significantly higher rate of ROSC was achieved in the CCSV + ABO group than the IPPV group. The combination of CCSV and ABO implemented during CPR significantly improved post-resuscitation myocardial and neurological dysfunction, decreased cardiac, cerebral, renal, intestinal injuries and their pathological damage, and increased the rate of survival when compared to the IPPV group.

The main goal of CPR performance is to optimize blood oxygenation and circulation after CA events, therefore maximize the prevention of organ damage and the achievement of ROSC, and furthermore improve post-resuscitation neurological outcome and the survival (15). Currently, the strategies of ventilation suggested by the CPR guidelines are to deliver IPPV with a 30:2 compression-ventilation ratio or without pausing chest compression (5, 16, 17); however, the former is performed using a bag respirator with room air during the interruption of chest compression (18), and the latter may cause the decrease in ventilation volume due to the potential resistance resulted from ongoing chest compression (19). Both of them could affect the supply of sufficient oxygen and the removal of carbon dioxide. In addition, high-quality manual, or alternative mechanical chest compression including adequate compression rate and depth, complete chest recoil, and minimized interruption was continued to be recommended by the latest guidelines (5, 16, 17); however, external chest compression can only provide less than 40% of normal blood flow to vital organs even when performed adequately (20). Thus, the techniques of CPR performance urgently need to be further optimized.

Chest compression synchronized ventilation, emerged as a short positive pressure ventilation mode exactly triggered at the beginning of each chest compression, has been shown to effectively avoid the ventilation problems mentioned above and generate significantly better oxygenation than the conventional IPPV mode (7, 8). In 2014, Kill et al. (7) demonstrated that CCSV elicited significantly higher PaO_2_ and lower PaCO_2_, and partly increased arterial blood pressure during CPR when compared with the IPPV mode; however, CCSV failed to improve the rate of ROSC when compared with IPPV. They also demonstrated that CCSV significantly improved arterial oxygenation and better maintained arterial blood pressure during CPR when compared with the IPPV mode (8). Recently, Speer et al. (21) demonstrated that CCSV delivered a significantly higher frequency of correct ventilation parameters without exceeding the upper pressure preset when compared with the IPPV mode during manual chest compression in a simulation model. In the present study, the same parameters of CCSV and IPPV were set based on the above-mentioned two studies, and the similar results were observed: CCSV significantly increased blood oxygenation, and partly improved blood circulation during CPR compared with the IPPV group. ROSC, post-resuscitation multiple organ injury, and survival were tended to be better in the CCSV group than in the IPPV group.

Aortic balloon occlusion, known as one kind of endovascular occlusion technique, has been confirmed to effectively control the hemorrhage of distal organs and increase the perfusion of proximal organs in patients with severe injuries (22). In the setting of hemorrhage-induced traumatic CA, we have demonstrated that ABO augmented the efficacy of CPR, and a 30-min occlusion improved post-resuscitation cardiac and cerebral outcomes without exacerbating renal and intestinal injuries (23). Recently, growing evidence suggests that ABO is also effective in improving systemic hemodynamics during CPR in the experimental setting and clinical cases of non-traumatic CA (24). In 2019, Tiba et al. (9) demonstrated that ABO significantly increased CPP and CBF during CPR and therefore achieved ROSC in 50% of swine. Dogan et al. (10) demonstrated that the ABO implemented in aortic zone I significantly increased systemic blood pressure and further enhanced visceral organ blood flow during CPR when compared with its implementation in aortic zone III or the control. Olsen et al. (11) employed a novel, automated ABO device, and demonstrated that ABO might increase cardiac and cerebral perfusion so as to protect the heart and brain during CPR and after ROSC. Hutin et al. (25) demonstrated that ABO was as effective as epinephrine to improve cardiac, cerebral, and systemic perfusion pressures, and avoided those potential cerebral impairment related to epinephrine administration. In the clinical setting, Gamberini et al. (26) demonstrated that ABO technique was feasible to perform as an adjunct to advanced life support in the pre-hospital setting and emergency room, in which most patients obtained a transient or sustained ROSC after balloon inflation. In the present study, ABO was placed in aortic zone I to perform endovascular occlusion during CPR based on those studies mentioned above. Consequently, the IPPV + ABO group improved systemic hemodynamics during CPR, and further alleviated post-resuscitation multiple organ injury compared with the IPPV group. However, this group did not make an improvement in ROSC, post-resuscitation myocardial and neurological dysfunction, and survival compared to the IPPV group.

Considering that the potential advantage and insufficiency of CCSV and ABO, the effectiveness of combined CCSV and ABO implemented during CPR was investigated in this study. The combination of CCSV and ABO simultaneously improved blood oxygenation and circulation during CPR, and further improved ROSC, post-resuscitation multiple organ injury, and survival compared with CCSV alone or IPPV + ABO. Most importantly, these improvements produced by combined CCSV and ABO were significantly greater compared to the IPPV group. Thus, the combined implementation of CCSV and ABO may become an effective strategy to enhance the efficacy of conventional CPR and further improve the patients’ outcomes.

There were some limitations in our study. First, blood oxygenation was significantly increased during CPR in those animals ventilated with the CCSV mode; however, this kind of high-frequency ventilation triggered by each chest compression resulted in the decrease of PaCO_2_, which might cause a detrimental effect on cerebral blood perfusion and oxygenation (27). Second, although combined CCSV and ABO improved CPR outcomes compared with CCSV alone or IPPV + ABO, the differences were not statistically significant with the small sample size and a moderate CA/CPR duration in our study. In addition, considering that the different sample sizes between the IPPV group and the other three groups might cause an impact on the actual results of the study, and a longer duration of CA/CPR would lead to a lower rate of ROSC and poorer survival in CA events, the effectiveness of CCSV and ABO combination needs to be further investigated in a longer duration of CA/CPR setting in the larger, same sample size of swine studies. Third, considering that the ABO implemented during CPR decreased aortic blood flow to the kidney and intestine, these two organs could not be protected during CPR in those animals receiving the ABO. Thus, the alleviation of renal and intestinal damage might be attributed to better blood perfusion and oxygenation, and milder systemic inflammation after resuscitation. The similar results were also observed in our previous study (23). However, their protective mechanisms still need to be investigated. Fourth, ABO implementation is an invasive intravascular procedure with a series of potential risks including bleeding, arterial injury, balloon rupture, cardiovascular collapse, hematoma, thromboembolism, etc. (28). Thus, its rapid, safe, and accurate placement during CPR would be a great challenge in the actual clinical setting. Currently, endovascular skills training, contrast-enhanced ultrasonography guidance, and smaller caliber of ABO device development have been shown to be helpful to implement the ABO technique (29–31). Fifth, the ABO implemented based on the preset pressure might be not enough to guarantee the complete occlusion of arterial blood flow in this study. It’s combination with the estimation of balloon inflation volume and the check of distal arterial pulse should be more accurate for the achievement of complete occlusion in the future. Sixth, although the same procedures of CA/CPR model were performed among the four groups in this animal study; however, their performance was not fully consistent with human CPR guidelines (5). Thus, this study’s applicability may be decreased for its translation to clinical practice.

## Conclusion

In the present study, mechanical ventilation with the CCSV mode facilitated blood oxygenation, and the ABO in aortic zone I strengthened blood circulation during CPR. Their combination significantly augmented the efficacy of CPR, and further resulted in a significant improvement in ROSC and post-resuscitation multiple organ injury in a swine model of CA and resuscitation.

## Data availability statement

The original contributions presented in this study are included in the article/supplementary material, further inquiries can be directed to the corresponding author.

## Ethics statement

The animal study was reviewed and approved by the Institutional Animal Care and Use Committee of the Second Affiliated Hospital, Zhejiang University School of Medicine.

## Author contributions

JX and MaZ designed the study. JX, ZK, MiZ, MeZ, and ZZ performed the experiments. JW, QC, GZ, and MaZ provided the materials. JX, ZK, MiZ, and ZZ performed the measurements and analyzed the data. JX wrote the manuscript. All authors contributed to the article and approved the submitted version.
