# Corrigendum: The combination of chest compression synchronized ventilation and aortic balloon occlusion improve the outcomes of cardiopulmonary resuscitation in swine

**DOI:** 10.3389/fmed.2023.1177034

**Published:** 2023-04-04

**Authors:** Jiefeng Xu, Zafar Ullah Khan, Minhai Zhang, Jiangang Wang, Meiya Zhou, Zhongjun Zheng, Qijiang Chen, Guangju Zhou, Mao Zhang

**Affiliations:** ^1^Department of Emergency Medicine, The Second Affiliated Hospital, Zhejiang University School of Medicine, Hangzhou, China; ^2^Key Laboratory of the Diagnosis and Treatment of Severe Trauma and Burn of Zhejiang Province, Hangzhou, China; ^3^Zhejiang Provincial Clinical Research Center for Emergency and Critical Care Medicine, Hangzhou, China; ^4^Hangzhou Emergency Medical Center, Hangzhou, China; ^5^Department of Intensive Care Medicine, The First Hospital of Ninghai, Ningbo, China

**Keywords:** aortic balloon occlusion, cardiac arrest, cardiopulmonary resuscitation, chest compression synchronized ventilation, hemodynamics, oxygenation, organ protection

In the published article, there was an error regarding the affiliations for Meiya Zhou. As well as having affiliation Hangzhou Emergency Medical Center, Hangzhou, China, she should also have the affiliation Department of Emergency Medicine, The Second Affiliated Hospital, Zhejiang University School of Medicine, Hangzhou, China.

The authors apologize for this error and state that this does not change the scientific conclusions of the article in any way. The original article has been updated.

